# Presence/Absence and Specific Location of Resident CD34+ Stromal Cells/Telocytes Condition Stromal Cell Development in Repair and Tumors

**DOI:** 10.3389/fcell.2020.544845

**Published:** 2020-09-18

**Authors:** Lucio Díaz-Flores, Ricardo Gutiérrez, Ma Pino García, Miriam González-Gómez, Lucio Díaz-Flores, Hugo Álvarez-Argüelles, José Luis Carrasco

**Affiliations:** ^1^Department of Basic Medical Sciences, Faculty of Medicine, University of La Laguna, Tenerife, Spain; ^2^Department of Pathology, Eurofins^®^ Megalab–Hospiten Hospitals, Tenerife, Spain

**Keywords:** gallbladder, colon, repair, tumor stroma, CD34+ stromal cells/telocytes

## Abstract

CD34+ stromal cells/telocytes (CD34+SCs/TCs) can have a role as mesenchymal precursor cells. Our objective is to assess whether the myofibroblastic stromal cell response in repair and in desmoplastic reactions in tumors depend on the presence or absence of resident CD34+SCs/TCs in specific regions/layers of an organ and on the location of their possible subpopulations. For this purpose, using conventional and immunohistochemical procedures, we studied specimens of (a) acute cholecystitis, with early repair phenomena (n: 6), (b) surgically resected segments of colon tattooed with India ink during previous endoscopic removal of malignant polyps, with macrophage infiltration and stromal cell reaction (n: 8) and (c) infiltrative adenocarcinomas of colon, with desmoplastic reaction (n: 8). The results demonstrated (a) stromal myofibroblastic reaction during repair and tumor desmoplasia in most regions in which resident CD34+SCs/TCs are present, (b) absence of stromal myofibroblastic reaction during repair in the mucosa of both organs in which resident CD34+SCs/TCs are absent and (c) permanence of CD34+SCs/TCs as such, without myofibroblastic response, in smooth muscle fascicles, nerves, and Meissner and Auerbach plexuses, in which the CD34+SCs/TCs mainly undergo reactive phenomena. Therefore, the development of activated αSMA+ myofibroblasts in these conditions requires the presence of resident CD34+SCs/TCs and depends on their location. In conclusion, the facts support the hypotheses that CD34+SCs/TCs participate in the origin of myofibroblasts during repair and tumor stroma formation, and that there is a heterogeneous population of resident CD34+SCs/TCs with different roles.

## Introduction

CD34+ stromal cells are present in the connective tissue of multiple anatomical sites. A new cellular type named telocyte was identified by electron microscopy and described in 2010 (Popescu and Faussone-Pellegrini, 2010; Faussone Pellegrini and Popescu, 2011; Popescu, 2011). This cell type largely corresponds to the CD34+ stromal cells observed in light microscopy (Rusu et al., 2016; Manetti et al., 2019). Several roles have been hypothesized for CD34+ stromal cells/telocytes (CD34+SCs/TCs) in (a) the maintenance and modulation of tissue homeostasis, including organization and control of the extracellular matrix, structural support, creation of microenvironments, integration of tissue components, control and regulation of other cells, and immunomodulation (Faussone Pellegrini and Popescu, 2011; Popescu, 2011; Popescu and Nicolescu, 2013; Díaz-Flores et al., 2014, 2016a; Cretoiu et al., 2016, 2020; Vannucchi et al., 2016) regeneration and repair (Faussone Pellegrini and Popescu, 2011; Popescu, 2011; Popescu et al., 2011; Ceafalan et al., 2012; Bani and Nistri, 2014; Díaz-Flores et al., 2015a,b, 2016b; Vannucchi et al., 2016). In regeneration, CD34+SCs/TCs act on neighboring parenchymal stem cells, regulating their proliferation and maturation (Faussone-Pellegrini and Bani, 2010; Popescu, 2011; Ceafalan et al., 2012; Gherghiceanu and Popescu, 2012; Rusu et al., 2016; Vannucchi and Faussone-Pellegrini, 2016; Cretoiu et al., 2017; Manetti et al., 2019). In repair through granulation tissue, CD34+SCs/TCs play an important role as precursor cells. Thus, they can change their location, relationship, proliferative activity, morphology, and immunohistochemical profile. For example, they can lose CD34 expression and gain alpha-smooth muscle actin (αSMA) expression, acquiring myofibroblastic characteristics (Nakayama et al., 2000, 2001, 2003; Barth et al., 2002a,b, 2004, 2005; Chauhan et al., 2003; Ramaswamy et al., 2003; Kuroda et al., 2005a,c; Ebrahimsade et al., 2007; Nimphius et al., 2007; Wessel et al., 2008; Díaz-Flores et al., 2015a,b, 2016b). The vascular/perivascular niche (da Silva Meirelles et al., 2006, 2015, 2016; Díaz-Flores et al., 2015a,b; Corrêa Bellagamba et al., 2018) is one of the principal sources of mesenchymal precursor cells that participate in tissue repair. Among these precursor cells are CD34+SCs/TCs, which are located around the abluminal surface of the pericytes and of the vascular smooth muscle cells. Thus, the CD34-stained vessels show a double-ring appearance (CD34+ endothelium and CD34+SCs/TCs), owing to two concentric circles “sandwiching” the unstained media layer (smooth muscle or pericytic layer) (Pusztaszeri et al., 2006; Lin et al., 2008, 2010; Díaz-Flores et al., 2015a,b).

Therefore, CD34+SCs/TCs can have multiple functions, which may depend on the location of their subpopulations (region-specific roles) (Pieri et al., 2008; Vannucchi et al., 2013; Díaz-Flores et al., 2016a; Cretoiu et al., 2017). In addition, they can be absent in some vessels and some regions or layers of an organ. Consequently, studying the stromal cell population (CD34+SCs/TCs and myofibroblasts) during repair, including tumor stroma formation as a special type of repair, in different areas of an organ, can reveal whether the response depends on (a) the regional presence or absence of CD34+SCs/TCs (general role of CD34+SCs/TCs in repair) and (b) the area in which these cells are located (different role of CD34+SCs/TCs subpopulations depending on location).

Given the above, the objective of this work is to assess whether the response of the stromal cell population in repair through granulation tissue, including tumor stromal formation, depends on the presence or absence of CD34+SCs/TCs and on their location within the organ. For this purpose, we study (1) the normal distribution of CD34+SCs/TCs in the different layers of the gallbladder and colon, and (2) the stromal cell response during repair in these organs, including the following processes (a) acute cholecystitis with initial and specific repair phenomena, (b) surgically resected segments of the colon after endoscopic resection of malignant polyps and India ink tattoos, leading to macrophage infiltration and stromal cell reaction, and (c) infiltrating colon adenocarcinomas with an important stromal reaction (desmoplastic reaction).

## Materials and Methods

### Human Tissue Samples

The archives of Histology and Anatomical Pathology of the Departments of Basic Medical Sciences of La Laguna University, University Hospital, and Eurofins^®^ Megalab–Hospiten Hospitals of the Canary Islands were searched for cases presenting the process outlined above, of which demonstrative samples were selected, including acute cholecystitis with initial and specific repair phenomena (n: 6), surgically resected segments of colon, following previous resection of malignant polyps (Parra-Blanco et al., 2006), tattooed with India ink (n: 8) and infiltrative adenocarcinomas of colon (n: 8), with later repair phenomena. In addition to confirming the results in different conditions and organs, an important objective is therefore to confirm the facts over time. Non-affected gallbladders and regions of the colon without pathologic findings (colectomies with a margin longer than 10 cm from the lesion) were also selected. Ethical approval for this study was obtained from the Ethics Committee of La Laguna University (Comité de Ética de la Investigación y de Bienestar Animal, CEIBA 2020-0382), including the dissociation of the samples from any information that could identify the patient. The authors therefore had no access to identifiable patient information.

### Light Microscopy

Specimens for conventional light microscopy were fixed in a buffered neutral 4% formaldehyde solution, embedded in paraffin and cut into 3 μm-thick sections. Sections were stained with Hematoxylin–Eosin (HE), PAS–Alcian Blue and Masson trichrome.

### Immunohistochemistry

Histologic sections, 3 μm-thick, were attached to silanized slides. After pre-treatment for enhancement of labeling, the sections were blocked with 3% hydrogen peroxide and then incubated with primary antibodies (10–40 min). The primary antibodies (Dako, Glostrup, Denmark) used in this study were CD34 monoclonal mouse anti-human, clone QBEnd-10, (dilution 1:50), catalog No. IR632, αSMA monoclonal mouse anti-human, clone 1A4 (dilution 1:50), catalog No. IR611 and podoplanin monoclonal mouse anti-human, clone D2-40 (dilution 1:50), catalog No. M3619. The immunoreaction was developed in a solution of diaminobenzidine and the sections were then briefly counterstained with hematoxylin, dehydrated in ethanol series, cleared in xylene and mounted in Eukitt^®^. Positive and negative controls were used. For the double immunostaining, we used anti-CD34 or anti-podoplanin antibodies (diaminobenzidine, DAB, as chromogen) and anti-αSMA (aminoethylcarbazole, AEC, substrate–chromogen).

### Immunofluorescence in Confocal Microscopy

For immunofluorescence, tissue sections were obtained as described above. For antigen retrieval, sections were deparaffinized and boiled for 20 min in sodium citrate buffer 10 mM (pH 6), rinsed in *Tris*-buffered saline (TBS, pH 7.6, 0.05 M), and incubated with the following primary antibodies diluted in TBS overnight in a humid chamber at room temperature: mouse monoclonal anti-CD34, code no. IR63261, Dako (ready to use), and goat polyclonal anti-actin (1/50 dilution, C-11, sc-1615, Santa Cruz Biotechnology). For the double immunofluorescence staining, sections were incubated with the mixture of monoclonal and polyclonal primary antibodies (mouse monoclonal anti-CD34 and goat polyclonal anti-actin). The next day, the slides were rinsed in TBS and incubated for 1 h at room temperature in the dark with the secondary biotinylated goat anti-rabbit IgG (H+L) (1:500, Code: 65-6140, Invitrogen, San Diego, CA, United States) and Alexa Fluor 488 goat anti-mouse IgG (H+L) antibody (1:500, Code: A11001, Invitrogen), followed by incubation with Streptavidin Cy3 conjugate (1:500, Code: SA1010, Invitrogen) for 1 h at room temperature in the dark. Nuclei were detected by DAPI staining (Chemicon International, Temecula, CA, United States). After washing in TBS, sections were exposed to a saturated solution of Sudan black B (Merck, Barcelona, Spain) for 20 min to block autofluorescence. They were rinsed in TBS and cover-slipped with DABCO (1%) and glycerol-PBS (1:1). Negative controls were performed in the absence of primary antibodies. Fluorescence immunosignals were obtained using a Fluoview 1000 laser scanning confocal imaging system (Olympus Optical).

## Results

### CD34+SCs/TCs in the Normal Gallbladder

**FIGURE 1:**
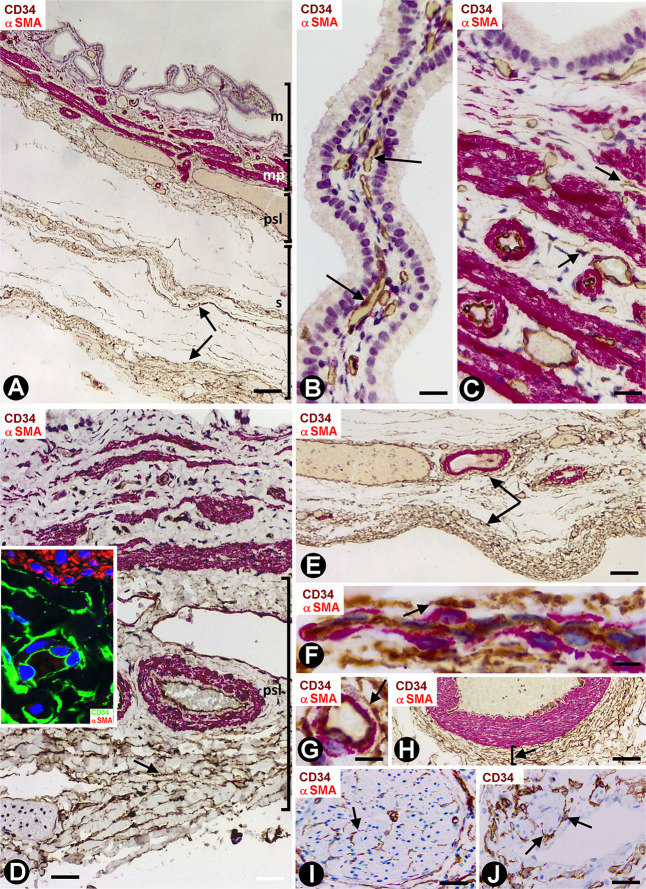
CD34+ SCFTs in the normal gallbladder. **(A–I)** Sections double-stained with anti-CD34 (brown) and anti-αSMA (red). **(J)** Section stained with anti-CD34 (brown). **(A)** Panoramic view of gallbladder wall layers, mucosa (m), muscularis propria (mp) (smooth muscle cells: red), perimuscular subserosal layer (psl) and serosa (s), in which a different distribution of CD34+SCs/TCs (brown) is observed, predominantly in the perimuscular subserosal layer and in strands in the serosa (arrows), parallel to the serosa surface. **(B)** Absence of CD34+SCs/TCs in the corium of the mucosa; the endothelial cells are the only cells stained with anti-CD34 (arrows). Note that CD34+SCs/TCs are also absent in the perivascular spaces, which may or may not contain pericytes. **(C,D)** CD34+SCs/TCs (arrows) in the muscularis propria. In panel **(D)**, observe numerous CD34+SCs/TCs (brown, arrow) in the perimuscular subserosa layer (psl). In the insert, smooth muscle cells (αSMA+, red) and CD34+SCs/TCs (green). **(E)** Abundant CD34+SCs/TCs (brown, arrows) in parallel strands in the serosa. **(F–J)** Details of the location of CD34+SCs/TCs in vessels, nerves, and lymphatics (arrows). Note CD34+SCs/TCs around pericytes in the pericytic microvasculature (**F,G**, arrows), in the adventitia of an artery (**H**, arrow), in a nerve (**I**, arrow) and in a lymphatic pre-collector (**J**, arrow). Bar: **(A)** 160 μm; **(B)** 20 μm; **(C,H–I)** 40 μm; **(D)** 60 μm; **(E)** 80 μm; **(F,G)** 10 μm.

Depending on the gallbladder layer (Figure 1A), the distribution of CD34+SCs/TCs was as follows: (1) absence in the lamina propria of the mucosa (Figure 1B) (although some of these cells were observed underlying the glands in the gallbladder neck), (2) presence in variable numbers in the other layers (Table 1), including the fibrovascular connective tissue that separates the muscle bundles of the muscularis propria (Figures 1C,D and insert), the perimuscular subserosal layer of connective tissue (Figure 1D and insert) and the serosa, in which CD34+SCs/TCs are generally arranged in strands parallel to the serosa surface (Figure 1E). When present, CD34+SCs/TCs were located in the perivascular niche (around pericytes) (Figures 1F,G), in the adventitia of greater vessels (around vascular smooth muscle cells) (Figure 1H), in the connective interstitium, and around and within nerves (Figure 1I). They were also seen in contact with the endothelium or smooth muscle cells in lymphatic vessels, including capillaries, pre-collectors (Figure 1J) and collectors, which were relatively frequent in the gallbladder serosa.

**TABLE 1:** Presence/Absence and semiquantification of CD34+SCs/TCs in normal conditions, and CD34+SCs/TCs and αSMA+cells (myofibroblasts) during repair and tumoral stroma formation.

	Gallbladder			Colon				
	Normal conditions	Repair in cholecystitis		Normal conditions	Repair in surgical resected segments after malignant polyps removed and India ink tattoo		Desmoplastic reaction in Invasive adenocarcinomas of colon	
					
	CD34+SCs/TCs	CD34+SCs/TCs	αSMA+ cells (myofibroblasts)	CD34+SCs/TCs	CD34+SCs/TCs	αSMA+ cells (myofibroblasts)	CD34+SCs/TCs	αSMA+ cells (myofibroblasts)
Mucosa of both organs	−	−	−	−	−	−	−	+/−
Perimuscular subserosal layer of gallbladder Submucosa of colon	++	−	+++	+++	−−	+++	−	+++
Muscularis propria	++/−	++	−	++/−	++	−	++	−−
Adventitia of arteries	++	++	+/−	++	++	+/−	++	+/−
Nerves in bath organs Meissner and myenteric plexuses in colon	++	+++	−	++	+++	−	+++	−
Serosa	+++/−	−	+++	+++/−	−	+++	−	+++

### Stromal Cells During Repair in Acute Cholecystitis

**FIGURE 2:**
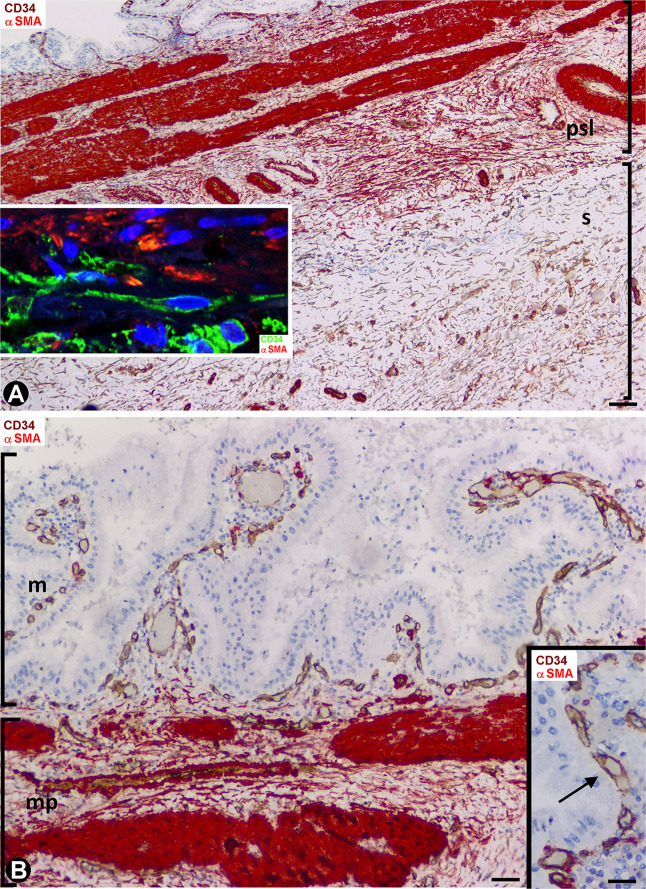
Stromal cells during repair in acute cholecystitis. Double staining with anti-CD34 (brown) and anti-αSMA (red). **(A)** Panoramic view of the gallbladder wall showing a high αSMA expression (deep red) in smooth muscle cells of the lamina propria and of vascular media layer, and moderate expression in myofibroblasts (numerous in the perimuscular serosal layer: psl). Note persistence of CD34+SCs/TCs (brown) in the area of serosa underlying the perimuscular serosa layer. In the insert, a zone of transition between αSMA+ cells (myofibroblasts, red) and CD34+SCs/TCs (green) is shown by fluorescent immunostaining in confocal microscopy. **(B)** Absence of activated stromal cells in the mucosa, whereas αSMA+ activated stromal cells (red) are present between the muscle fascicles (deep red) of the muscularis propria. In the insert, detail of the vessels (arrows) in the mucosa. Bar: **(A)** 60 μm, **(B)** 40 μm; Insert: 20 μm.

**FIGURE 3:**
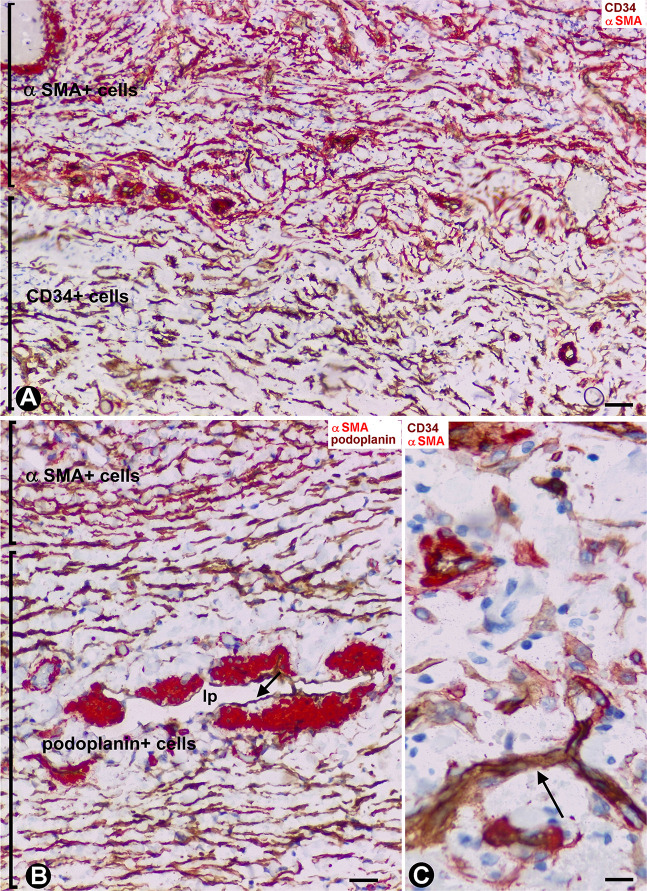
Stromal cells during repair in acute cholecystitis. **(A,C)** Double staining with anti-CD34 (brown) and anti-αSMA (red). **(B)** Double staining with anti-αSMA and anti-podoplanin. **(A)** αSMA+ activated stromal cells (myofibroblastic phenotype, red) observed in the perimuscular subserosal layer, alternating with a subjacent band of CD34+ activated stromal cells (brown). **(B)** αSMA activated stromal cells of a strand of the serosal layer are observed alternating with a band of podoplanin+ cells, which also express CD34 (not shown). Note the presence of a lymphatic pre-collector vessel (LpV) with podoplanin+ endothelial cells (arrow) and groups of SMCs (red) in its wall. **(C)** Cells expressing CD34 (brown) and αSMA (red). A capillary is also observed (arrow) Bar: **(A,B)** 40 μm; **(C)** 10 μm.

In general, the gallbladders with acute cholecystitis showed findings of early repair, including vasodilation, edema, angiogenic phenomena, margination, extravasation and infiltration of the leukocytes, and an increase in the number, size and characteristics of stromal cells (hereinafter activated stromal cells) (Figure 2A). These activated stromal cells were either CD34+ (CD34+SCs/TCs) or αSMA+ (myofibroblasts) (Figure 2A and insert). However, the mucosa, when conserved, did not show activated stromal cells (Figure 2B). An important population of activated αSMA+ CD34- stromal cells was observed in the fibrovascular connective tissue that separates the muscle bundles in the muscularis propria (Figure 2) and in the perimuscular subserosal layer of connective tissue (Figures 2A, 3A). Therefore, loss of CD34 expression and gain of αSMA expression (myofibroblastic phenotype) occurred in these areas. In the serosa layer, increased CD34+ stromal cells were observed underlying the perimuscular subserosal layer (Figure 3A) and between the strands described in normal conditions. Conversely, the perimuscular subserosal layer (as aforementioned) and the strands presented numerous αSMA+ stromal cells (myofibroblastic phenotype). Thus, bands of cells expressing CD34 or αSMA were seen between the subserosal limit of the muscularis propria and the serosal surface. The weak expression of podoplanin that CD34+SCs/TCs normally present became occasionally intense in stromal cells (Figure 3B) which conserved their CD34 positivity. Abundant reactive CD34+SCs/TCs were observed in the nerves. Occasionally, cells expressing both markers were seen (Figure 3C).

### CD34+SCs/TCs in the Normal Intestinal Wall

**FIGURE 4:**
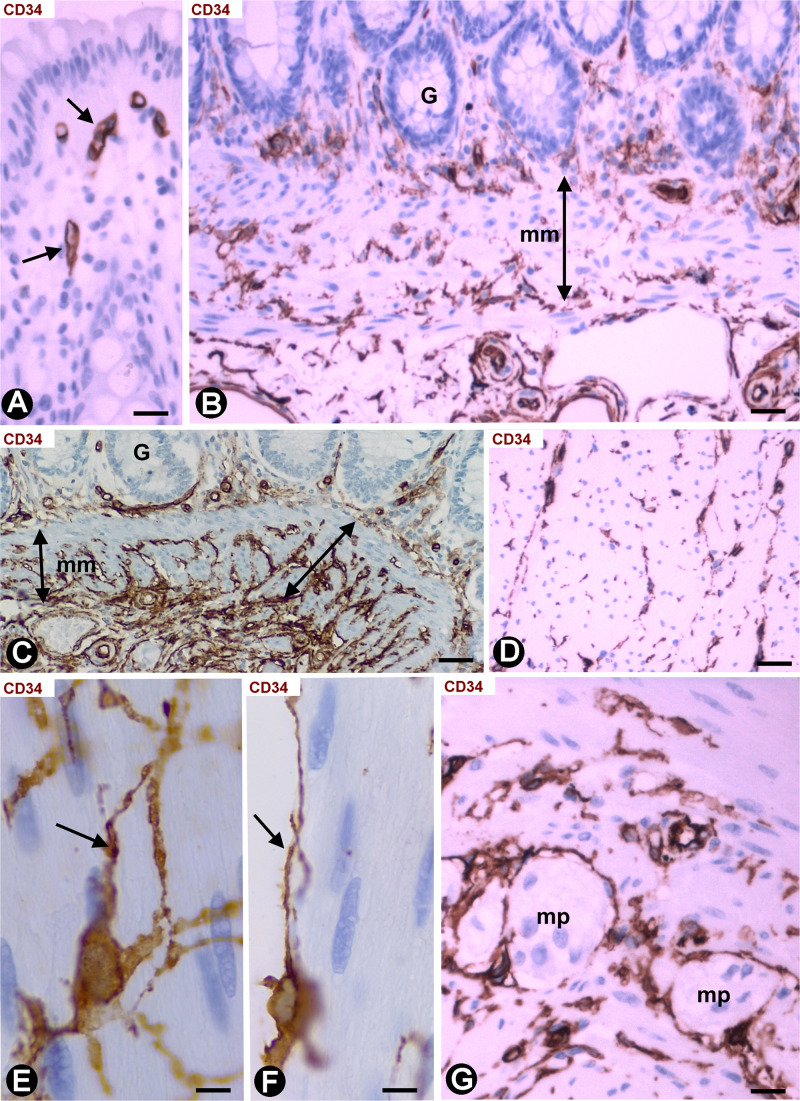
CD34+SCs/TCs in the normal intestinal wall. Sections stained with anti-CD34. **(A)** Absence of CD34+SCs/TCs in the chorion of the mucosa underlying the epithelium (only the endothelial cells express CD34, arrow). **(B,C)** Varying numbers of CD34+SCs/TCs (brown) in the areas between the base of the mucosa tubular glands and the muscularis mucosae (mm). A portion of the submucosa with abundant CD34+SCs/TCs (brown) is also observed in panel **(C)**. **(D)** CD34+SCs/TCs (brown) are seen within and around the muscle fascicles of the muscularis propria. **(E,F)** Details of the CD34+SCs/TCs within (**E**, arrow) and around (**F**, arrow) the muscle fascicles. **(G)** CD34+SCs/TCs in myenteric plexuses (mp). Bar: **(A,G)** 20 μm; **(B–D)** 40 μm; **(E,F)** 10 μm.

Depending on the intestinal layer, the CD34+SCs/TCs showed the following distribution: (1) Absence in the mucosa (Figure 4A), except in the areas underlying the tubular glands and the muscularis mucosae, where they were present in variable numbers (Figures 4B,C). (2) Presence in variable numbers (Table 1) in (a) the submucosa (Figure 4C), including perivascular niches (around the pericytes), the adventitia of the largest vessels (around vascular smooth muscle cells), the interstitium, the nerves and the Meissner plexus, (b) the muscularis propria, including the muscle fascicles (within and around them) (Figures 4D–F), nerves and the myenteric plexus (Figure 4G), close to the c-kit+ interstitial cells of Cajal, and (c) the serosa, including perivascular niches, adventitia of larger vessels, interstitium, nerves and adipose tissue.

### Stromal Cells in Segments of Colon Tattooed With India Ink, Following Previous Resection of Malignant Polyps

**FIGURE 5:**
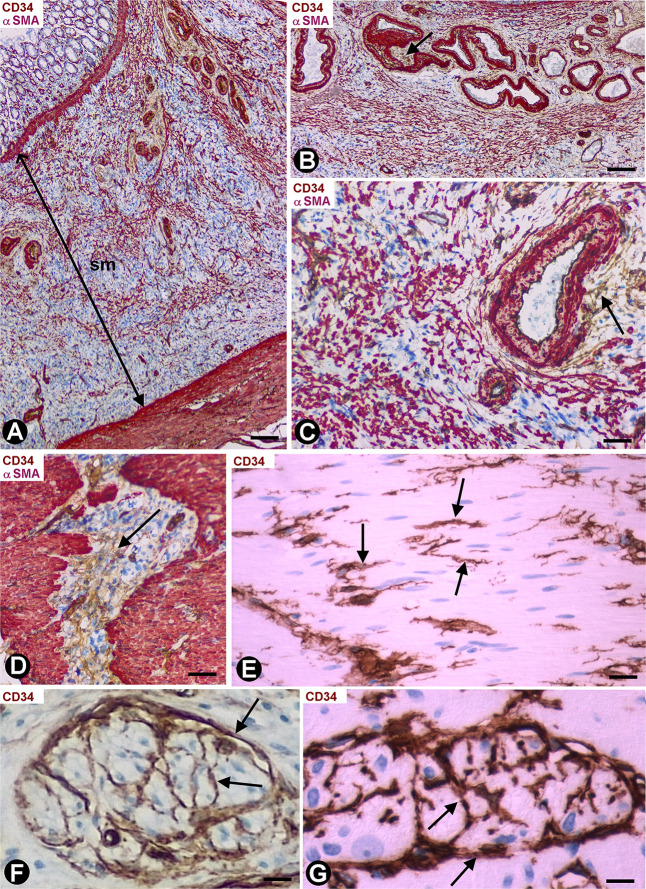
Stromal cells in segments of colon following resection of malignant polyps, tattooed with India ink, and with an intense infiltrate of macrophages. **(A)** Activated stromal cells are present in the submucosa and absent in the mucosa. Note that the activated stromal cells in the submucosa (sm) are αSMA+ (red). Infiltrates of macrophages are also observed in the mucosa and submucosa. **(B,C)** Absence of stromal cell reaction in the adventitia of large submucosal vessels, in which the CD34+SCs/TCs (brown, arrows) are conserved. **(D,E)** Activated CD34+SCs/TCs (brown) in the connective tissue between (**D**, arrow) and within (**E**, arrows) muscle fascicles. **(F,G)** Presence of CD34+SCs/TCs (brown) in a nerve (**F**, arrows) and in a myenteric ganglion (**G**, arrows). **(A–D)** Double staining with anti-CD34 (brown) and anti-SMA (red). **(E–G)** Sections stained with anti-CD34. Bar: **(A)** 60 μm; **(B)** 40 μm; **(C–F)** 20 μm; **(G)** 15 μm.

We observed an intense infiltrate of macrophages, isolated or forming small groups, and varying numbers (Table 1) of activated stromal cells in the segments of colon, following resection of malignant polyps tattooed with India ink. In the mucosa, the stromal cell reaction was not evident (Figure 5A). Stromal cells expressing αSMA (myofibroblastic phenotype) were observed between the macrophages in the submucosa (Figures 5A–C) and serosa, CD34+SCs/TCs were not observed in these layers. However, activated stromal cells remained positive for anti-CD34 and were negative for anti-αSMA in the adventitia of sub-mucosal and serosal medium sized vessels (Figures 5B,C), and between (Figure 5D) and within (Figure 5E) the muscle fascicles, nerves (Figure 5F) and myenteric plexuses (Figure 5G) in the muscularis layer.

### Stromal Cells in Infiltrative Carcinomas of Colon

**FIGURE 6:**
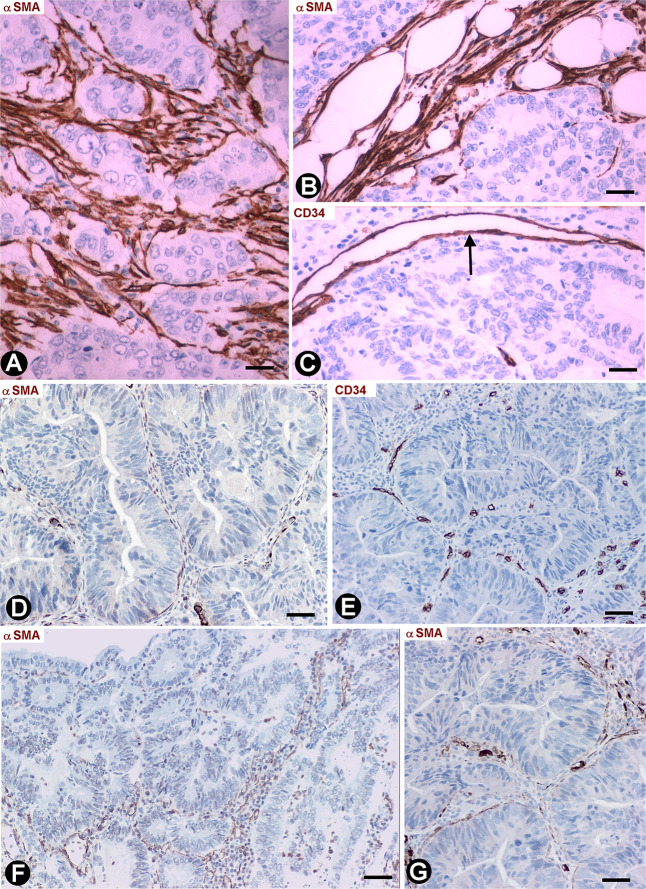
Stromal cells in infiltrative carcinomas of colon. **(A,B,D,F,G)** Sections stained with anti-αSMA. **(C,E)** Sections stained with anti-CD34. **(A,B)** αSMA+ stromal cells (brown) are observed around neoplastic glands in the submucosa **(A)** and serosa **(B)**. **(C)** Absence of CD34+SCs/TCs around neoplastic glands in the submucosa. Note that the endothelial cells are the only cells expressing CD34 (arrow). **(D–G)** Neoplastic glands in the mucosa. The stromal cell reaction is scarce and irregular with few αSMA+ cells (brown) **(D,F,G)**. CD34+SCs/TCs s are absent **(E)**. Bar: **(A)** 20 μm; **(B–G)** 40 μm.

**FIGURE 7:**
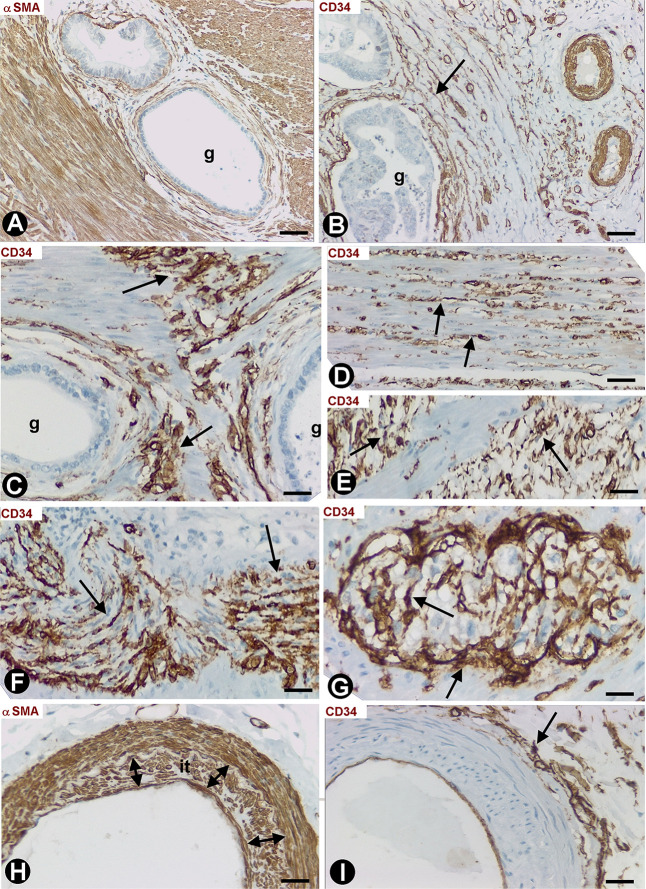
Stromal cells in infiltrative carcinomas of colon. **(A,H)** Sections stained with anti-αSMA. **(B–G,I)** Sections stained with anti-CD34. **(A)** Scarce αSMA+stromal cells (arrow) around neoplastic glands (g) present in the connective tracts between the muscle fascicles of the muscularis propria. **(B,C)** Activated CD34+SCs/TCs (brown, arrow) neighboring neoplastic glands (g) in the muscularis propria. **(D–G)** activated CD34+SCs/TCs within (**D**, arrow) and between (**E**, arrow) muscle fascicles, a nerve (brown, arrow) and a myenteric plexus (brown, arrow). **(H)** Intimal thickening formed by αSMA+ cells is observed in an artery of the serosa. **(I)** Adventitial CD34+SCs/TCs (brown, arrow) are seen in an artery with intimal thickening. Bar: **(A–F,H,I)** 20 μm; **(G)** 10 μm.

Infiltrative adenocarcinomas of the colon showed a desmoplastic reaction in the submucosa (Figure 6A) and serosa (Figure 6B), in which numerous elongated stromal cells around the neoplastic glands presented αSMA expression (myofibroblastic phenotype) (Figures 6A,B) and were negative for anti-CD34 (Figure 6C). In the mucosa, the desmoplastic reaction was irregular and scarce (Figures 6D–G). Thus, activated αSMA+ stromal cells were absent in some regions of the mucosa (Figure 6D), whereas αSMA+ stromal cells were seen around the neoplastic glands in other regions (Figures 6F,G). CD34+SCs/TCs were also absent around the neoplastic glands in the mucosa (Figure 6E), which generally were in vicinity to fragmented muscularis mucosa. An irregular stromal reaction was also observed in the connective tracts between the muscle fascicles of the muscularis propria, with presence of activated cells showing αSMA (Figure 7A) and CD34 (Figures 7B,C) expression. Likewise, CD34+SCs/TCs were present in the muscle fascicles (Figures 7D,E), nerves (Figure 7F) and myenteric plexuses (Figure 7G). In the serosa, intimal thickening was also observed in medium-caliber arteries, with presence of αSMA myointimal cells (Figure 7H) and adventitial CD34+SCFTs (Figure 7I).

### Common Findings and Their Variations in the Histological Samples Evaluated

The data described were present in all histological samples in the groups studied except those of the gallbladder serosa with acute cholecystitis and the colon mucosa with invasive carcinoma. In four cases of gallbladder with acute cholecystitis, the response affected the entire serosa, and in the other two cases, it was zonal. In the colon mucosa with invasive adenocarcinoma, the stromal response was absent in four cases and scarce in the other four cases.

## Discussion

We report that the repair response in different processes of the gallbladder and colon, including stroma formation in tumors, depends on the regional presence or absence of CD34+SCs/TCs (there is no myofibroblast response where resident CD34+SCs/TCs are absent) and on their location within the organ (with or without myofibroblast response). These findings support the hypothesis that resident CD34+SCs/TCs play an important role as stromal precursor cells in tissue repair and tumor stroma formation, and that there is a heterogeneous population of resident CD34+SCs/TCs with different roles. We consider these issues below.

We have used the term CD34+SCs/TCs because telocytes identified in electron microscopy largely correspond to CD34+ stromal cells observed in light microscopy (Rusu et al., 2016; Manetti et al., 2019). However, further studies, including transmission electron microscopy observation, are required. Indeed, the advantage of immunohistochemistry in light microscopy is that it enables the observation of CD34+SC/TCs in extensive areas, while electron microscopy allows a better identification of telocytes.

Resident CD34+SCs/TCs are located in multiple anatomical sites, including vessels (around pericytes in pericytic microvasculature and in the adventitia of larger vessels), loose connective tissue, capsules, fibrous tracts, adipose tissue, cardiac, smooth and skeletal muscle, nerves, and around glands and skin annexes (Pieri et al., 2008; Bani et al., 2010; Popescu and Faussone-Pellegrini, 2010; Popescu et al., 2011; Nicolescu and Popescu, 2012; Nicolescu et al., 2012; Vannucchi et al., 2014; Díaz-Flores et al., 2016a). Conversely, resident CD34+SCs/TCs are not observed in some organic regions. Examples of organs with or without CD34+SCs/TCs, depending on the layer examined, are the gallbladder and the intestine, whose mucosa is devoid of CD34+SCs/TCs (except occasional presence between the base of glands and the muscularis propria in the gallbladder and the muscularis mucosae in the intestine), whereas CD34+SCs/TCs are present in the other layers. These observations in the intestine coincide with those of other authors (Nakayama et al., 2000: Pieri et al., 2008; Vannucchi et al., 2013). Hinescu et al. (2007) have described interstitial Cajal-like cells expressing CD117 and CD34 in the lamina propria of the gallbladder, which can also coincide with subepithelial cells in the base of the glands near the muscularis propria in the gallbladder neck.

Given the above, we chose the gallbladder and colon to study the CD34+SC/TC and myofibroblast response during repair for the following reasons: these organs contain layers with or without resident CD34+SC/TCs, with a well-known CD34+SC/TC distribution, above all in the intestine; and they present repair phenomena in certain diseases, which require surgical removal. Another advantage is knowing this response *in situ* and in humans. One disadvantage could be that the study is based on snapshot observations. However, the observation of repair phenomena at different stages (initial stage in the gallbladder and more advanced in the colon) has allowed us to confirm the facts over time. Indeed, in acute cholecystitis, whose treatment is usually the urgent laparoscopic removal of the gallbladder, the repair phenomena were at an early phase. Conversely, following resection of malignant polyps and India ink tattoo, repair was more advanced in the colon, in which no urgent secondary surgical intervention after pathological diagnosis is the treatment.

The data described were present in most of the histologic samples with exceptions. The different extension of the lesion in the serosa of the gallbladder may be related to the intensity of the process. The variable response in the intestinal mucosa affected by invasive adenocarcinoma will be discussed below.

That growth and development of activated αSMA+ myofibroblasts were lacking during repair in the mucosa of the gallbladder and colon, in which CD34+SCs/TCs are absent (Nakayama et al., 2000; Pieri et al., 2008; Vannucchi et al., 2013), shows that CD34+SCs/TCs are essential to this process of myofibroblast development, supporting the hypothesis that myofibroblasts in repair can originate from CD34+SCs/TCs (resident CD34+SDs/TCs as precursor cells) (Nakayama et al., 2001, 2002a,b; Barth et al., 2002a, 2005; Ramaswamy et al., 2003; Kuroda et al., 2005b; Ebrahimsade et al., 2007; Nimphius et al., 2007; Wessel et al., 2008; Díaz-Flores et al., 2015a,b, 2016b).

There are numerous studies about cells expressing CD34 with different phenotypes, mainly in bone marrow and blood samples (CD34+ hematopoietic cells). We have circumscribed our observations to resident CD34+SC/TC behavior during repair. The results demonstrate different responses of CD34+SCs/TCs, depending on their location, with myofibroblast growth and development and CD34+SCs/TC disappearance (e.g., submucosa and serosa of colon, and perimuscular serosal layer of gallbladder) or with no myofibroblast development and persistence of reactive CD34+SCs/TCs (e.g., nerves, ganglia and smooth muscle fascicles). This CD34+SC/TC behavior also supports the hypothesis of heterogeneous subpopulations of CD34+SCs/TCs with different roles (Pieri et al., 2008; Vannucchi et al., 2013; Cretoiu et al., 2017). Whether or not these cells with different roles have a different phenotype requires verification.

Interpretation of the stromal cell response in tumors depending on location is more difficult, since tumor type may also influence the response. Thus, although there is a phenotypic shift from CD34+SCs/TCs toward αSMA+ myofibroblasts in numerous malignant invasive epithelial tumors (Nakayama et al., 2000, 2001, 2003; Barth et al., 2002a,b, 2004, 2005; Chauhan et al., 2003; Ramaswamy et al., 2003; Kuroda et al., 2005a,c; Nimphius et al., 2007; Wessel et al., 2008), the reaction is not constant. For example, CD34+SCs/TCs are preserved (reactive stromal cells are CD34+) in most lobular carcinoma of the breast, with no transformation into reactive αSMA+ stromal cells (Ebrahimsade et al., 2007). For this reason, we selected invasive adenocarcinomas of the colon in which reactive αSMA+ stromal cells are always present in the layers beyond the muscularis mucosae (Nakayama et al., 2000).

Our results concur with the study of Nakayama et al. (2000) on the activated stromal cells (growth and development of myofibroblasts and loss of CD34+SCs/TCs) in colonic adenocarcinomas. However, when we considered the stromal response in specific locations in the colon wall, the permanence of CD34+SCs/TCs, without myofibroblastic transformation, is a constant phenomenon in the muscle fascicles, nerves, and Meissner and Auerbach plexuses. Therefore, the response in these specific locations is similar to that observed in the repair processes outlined above, also suggesting the heterogeneity of the CD34+SCs/TCs. Nevertheless, the presence or not of αSMA+ cells around neoplastic glands in the mucosa is more difficult to interpret. It could be due to the persistence of pericryptal cells, normally present in the intestinal mucosa, or to infiltration of neoplastic glands surrounded by αSMA+ cells from the submucosa, a question that requires further study.

An important finding in the colonic infiltrating carcinomas was the frequent intimal thickening in arteries located in the serosa, which were in proximity to but not in contact with neoplastic glands. The adventitia participates in artery remodeling (Shen et al., 2016) and the role of the adventitial CD34+SCs/TCs in the arterial intimal thickening in adenocarcinomas of colon also requires further study.

## Conclusion

We demonstrate that, during repair in the gallbladder and colon, αSMA+ myofibroblasts develop in areas in which resident CD34+SCs/TCs are present, but not where these cells are absent. Likewise, αSMA+ cells can or cannot develop depending on the regional-specific location of resident CD34+SCs/TCs. These findings support the hypotheses that myofibroblasts in repair originate from CD34+SCs/TCs and that subpopulations of CD34+SCs/TCs have different roles. Further studies are required in this field, including electron microscopy observation.

## Data Availability Statement

The raw data supporting the conclusions of this article will be made available by the authors, without undue reservation.

## Ethics Statement

Ethical approval for this study was obtained from the Ethics Committee of La Laguna University (Comité de Ética de la Investigación y de Bienestar Animal, CEIBA 2020-0382), including the dissociation of the samples from any information that could identify the patient. The authors therefore had no access to identifiable patient information.

## Author Contributions

LD-F designed the study, acquired, analyzed and interpreted the data, and drafted and revised the manuscript. RG contributed to the study design, analyzed and interpreted the data, and drafted and revised the manuscript. MG analyzed and interpreted the data. MG-G interpreted immunofluorescence in confocal microscopy. LD-F Jr., HA-A, and JC analyzed and interpreted the data and revised the manuscript. All authors listed have reviewed and approved the final version of the manuscript for submission.

## Conflict of Interest

The authors declare that the research was conducted in the absence of any commercial or financial relationships that could be construed as a potential conflict of interest.

## References

[B1] Bani D. Formigli L. Gherghiceanu M. Faussone-Pellegrini M. S. (2010). Telocytes as supporting cells for myocardial tissue organization in developing and adult heart. *J. Cell Mol. Med.* 14 2531–2538. 10.1111/j.1582-4934.2010.01119.x 20977627 PMC3823169

[B2] Bani D. Nistri S. (2014). New insights into the morphogenic role of stromal cells and their relevance for regenerative medicine. Lessons from the heart. *J. Cell Mol. Med.* 18 363–370. 10.1111/jcmm.12247 24533677 PMC3955144

[B3] Barth P. J. Moll R. Ramaswamy A. (2005). Stromal remodeling and SPARC (secreted protein acid rich in cysteine) expression in invasive ductal carcinomas of the breast. *Virch. Arch.* 446 532–536. 10.1007/s00428-005-1256-9 15838642

[B4] Barth P. J. Ebrahimsade S. Ramaswamy A. Moll R. (2002a). CD34+ fibrocytes in invasive ductal carcinoma, ductal carcinoma in situ, and benign breast lesions. *Virch. Arch.* 440 298–303. 10.1007/s004280100530 11889601

[B5] Barth P. J. Ramaswamy A. Moll R. (2002b). CD34(+) fibrocytes in normal cervical stroma, cervical intraepithelial neoplasia III, and invasive squamous cell carcinoma of the cervix uteri. *Virch. Arch.* 441 564–568. 10.1007/s00428-002-0713-y 12461613

[B6] Barth P. J. Schenck zu Schweinsberg T. Ramaswamy A. Moll R. (2004). CD34+ fibrocytes, alpha-smooth muscle antigen-positive myofibroblasts, and CD117 expression in the stroma of invasive squamous cell carcinomas of the oral cavity, pharynx, and larynx. *Virch. Arch.* 444 231–234. 10.1007/s00428-003-0965-1 14758552

[B7] Ceafalan L. Gherghiceanu M. Popescu L. M. Simionescu O. (2012). Telocytes in human skin–are they involved in skin regeneration? *J. Cell Mol. Med.* 16 1405–1420. 10.1111/j.1582-4934.2012.01580.x 22500885 PMC3823211

[B8] Chauhan H. Abraham A. Phillips J. R. Pringle J. H. Walker R. A. Jones J. L. (2003). There is more than one kind of myofibroblast: analysis of CD34 expression in benign, in situ, and invasive breast lesions. *J. Clin. Pathol.* 56 271–276. 10.1136/jcp.56.4.271 12663638 PMC1769930

[B9] Corrêa Bellagamba B. Grudzinski P. B. Ely P. B. Nader P. J. H. Nardi N. B. da Silva Meirelles L. (2018). Induction of expression of CD271 and CD34 in mesenchymal stromal cells cultured as spheroids. *Stem Cells Int.* 2018:7357213. 10.1155/2018/7357213 30154865 PMC6091361

[B10] Cretoiu D. Radu B. M. Banciu A. Banciu D. D. Cretoiu S. M. (2017). Telocytes heterogeneity: From cellular morphology to functional evidence. *Semin. Cell. Dev. Biol.* 64 26–39. 10.1016/j.semcdb.2016.08.023 27569187

[B11] Cretoiu D. Roatesi S. Bica I. Plesca C. Stefan A. Bajenaru O. (2020). Simulation and modeling of telocytes behavior in signaling and intercellular communication processes. *Int. J. Mol. Sci.* 21 2615. 10.3390/ijms21072615 32283771 PMC7177713

[B12] Cretoiu D. Xu J. Xiao J. Cretoiu S. M. (2016). Telocytes and their extracellular vesicles-evidence and hypotheses. *Int. J. Mol. Sci.* 17 1322. 10.3390/ijms17081322 27529228 PMC5000719

[B13] da Silva Meirelles L. Bellagamba B. C. Camassola M. Nardi N. B. (2016). Mesenchymal stem cells and their relationship to pericytes. *Front. Biosci.* 21:130–156. 10.2741/4380 26709765

[B14] da Silva Meirelles L. Chagastelles P. C. Nardi N. B. (2006). Mesenchymal stem cells reside in virtually all post-natal organs and tissues. *J. Cell. Sci.* 119 2204–2213. 10.1242/jcs.02932 16684817

[B15] da Silva Meirelles L. Malta T. M. Panepucci R. A. da Silva W. A. Jr. (2015). Transcriptomic comparisons between cultured human adipose tissue-derived pericytes and mesenchymal stromal cells. *Genom. Data* 7 20–25. 10.1016/j.gdata.2015.11.009 26981353 PMC4778596

[B16] Díaz-Flores L. Gutiérrez R. Díaz-Flores L. Jr. Goméz M. G. Sáez F. J. Madrid J. F. (2016a). Behaviour of telocytes during physiopathological activation. *Semin. Cell Dev. Biol.* 55 50–61. 10.1016/j.semcdb.2016.01.035 26826526

[B17] Díaz-Flores L. Gutiérrez R. García M. P. González M. Díaz-Flores L. Madrid J. F. (2016b). Telocytes as a source of progenitor cells in regeneration and repair through granulation tissue. *Curr. Stem Cell Res. Ther.* 11 395–403. 10.2174/1574888x10666151001115111 26423297

[B18] Díaz-Flores L. Gutiérrez R. García M. P. Sáez F. J. Aparicio F. Díaz-Flores L. (2014). Uptake and intracytoplasmic storage of pigmented particles by human CD34+ stromal cells/telocytes: endocytic property of telocytes. *J. Cell Mol. Med.* 18 2478–2487. 10.1111/jcmm.12437 25266164 PMC4256559

[B19] Díaz-Flores L. Gutiérrez R. García M. P. González M. Sáez F. J. Aparicio F. (2015a). Human resident CD34+ stromal cells/telocytes have progenitor capacity and are a source of αSMA+ cells during repair. *Histol. Histopathol.* 30 615–627. 10.14670/HH-30.615 25500909

[B20] Díaz-Flores L. Gutiérrez R. Lizartza K. Goméz M. G. García M. D. P. Sáez F. J. (2015b). Behavior of in situ human native adipose tissue CD34+ stromal/progenitor cells during different stages of repair. Tissue-resident CD34+ stromal cells as a source of myofibroblasts. *Anat. Rec.* 298 917–930. 10.1002/ar.23086 25387858

[B21] Ebrahimsade S. Westhoff C. C. Barth P. J. (2007). CD34+ fibrocytes are preserved in most invasive lobular carcinomas of the breast. *Pathol. Res. Prac.* 203 695–698. 10.1016/j.prp.200 17656039

[B22] Faussone Pellegrini M. S. Popescu L. M. (2011). Telocytes. *Biomol. Concepts* 2 481–489. 10.1515/BMC.2011.039 25962049

[B23] Faussone-Pellegrini M. S. Bani D. (2010). Relationships between telocytes and cardiomyocytes during pre- and post-natal life. *J. Cell Mol. Med.* 14 1061–1063. 10.1111/j.1582-4934.2010.01074.x 20455994 PMC3822741

[B24] Gherghiceanu M. Popescu L. M. (2012). Cardiac telocytes - their junctions and functional implications. *Cell Tissue Res.* 348 265–279. 10.1007/s00441-012-1333-8 22350946 PMC3349856

[B25] Hinescu M. E. Ardeleanu C. Gherghiceanu M. Popescu L. M. (2007). Interstitial Cajal-like cells in human gallbladder. *J. Mol. Histol.* 38 275–284. 10.1007/s10735-007-9099-0 17541711

[B26] Kuroda N. Guo L. Miyazaki E. Hamauzu T. Toi M. Hiroi M. (2005a). The appearance of myofibroblasts and the disappearance of CD34-positive stromal cells in the area adjacent to xanthogranulomatous foci of chronic cholecystitis. *Histol. Histopathol.* 20 127–133. 10.14670/HH-20.127 15578431

[B27] Kuroda N. Jin Y. L. Hamauzu T. Toi M. Miyazaki E. Hiroi M. (2005b). Consistent lack of CD34-positive stromal cells in the stroma of malignant breast lesions. *Histol. Histopathol.* 20 707–712. 10.14670/HH-20.707 15944918

[B28] Kuroda N. Nakayama H. Miyazaki E. Toi M. Hiroi M. Enzan H. (2005c). The distribution of CD34-positive stromal cells and myofibroblasts in colorectal carcinoid tumors. *Histol. Histopathol.* 20 27–33. 10.14670/HH-20.27 15578419

[B29] Lin C. S. Xin Z. C. Deng C. H. Ning H. Lin G. Lue T. F. (2010). Defining adipose tissue-derived stem cells in tissue and in culture. *Histol. Histopathol.* 25 807–815. 10.14670/HH-25.807 20376787

[B30] Lin G. Garcia M. Ning H. Banie L. Guo Y. L. Lue T. F. (2008). Defining stem and progenitor cells within adipose tissue. *Stem Cells Dev.* 17 1053–1063. 10.1089/scd.2008.0117 18597617 PMC2865901

[B31] Manetti M. Tani A. Rosa I. Chellini F. Squecco R. Idrizaj E. (2019). Morphological evidence for telocytes as stromal cells supporting satellite cell activation in eccentric contraction-induced skeletal muscle injury. *Sci. Rep.* 9:14515. 10.1038/s41598-019-51078-z 31601891 PMC6787026

[B32] Nakayama H. Enzan H. Miyazaki E. Kuroda N. Naruse K. Hiroi M. (2000). Differential expression of CD34 in normal colorectal tissue, peritumoral inflammatory tissue, and tumour stroma. *J. Clin. Pathol.* 53 626–629. 10.1136/jcp.53.8.626 11002768 PMC1762933

[B33] Nakayama H. Enzan H. Miyazaki E. Kuroda N. Naruse K. Kiyoku H. (2001). CD34 positive stromal cells in gastric adenocarcinomas. *J. Clin. Pathol.* 54 846–848. 10.1136/jcp.54.11.846 11684718 PMC1731305

[B34] Nakayama H. Enzan H. Miyazaki E. Moriki T. Toi M. Zhang Y. (2002a). CD34-positive stromal cells and alpha-smooth muscle actin-positive stromal cells in the tumor capsule of skin sweat gland neoplasms. *Pathol. Int.* 52 25–30. 10.1046/j.1440-1827.2002.01317.x 11940203

[B35] Nakayama H. Enzan H. Miyazaki E. Toi M. (2002b). Alpha smooth muscle actin positive stromal cells in gastric carcinoma. *J. Clin. Pathol.* 55 741–744. 10.1136/jcp.55.10.741 12354798 PMC1769785

[B36] Nakayama H. Enzan H. Yamamoto M. Miyazaki E. Hidaka C. Okumichi T. (2003). CD34-positive stromal cells in primary lung carcinomas. *Oncol. Rep.* 10 1313–1316. 12883699

[B37] Nicolescu M. I. Bucur A. Dinca O. Rusu M. C. Popescu L. M. (2012). Telocytes in parotid glands. *Anat. Rec.* 295 378–385. 10.1002/ar.21540 22174191

[B38] Nicolescu M. I. Popescu L. M. (2012). Telocytes in the interstitium of human exocrine pancreas: ultrastructural evidence. *Pancreas* 41 949–956. 10.1097/MPA.0b013e31823fbded 22318257

[B39] Nimphius W. Moll R. Olbert P. Ramaswamy A. Barth P. J. (2007). CD34+ fibrocytes in chronic cystitis and noninvasive and invasive urothelial carcinomas of the urinary bladder. *Virch. Arch.* 450 179–185. 10.1007/s00428-006-0347-6 17149610

[B40] Parra-Blanco A. Gimeno-García A. Z. Nicolás-Pérez D. García C. Medina C. Díaz-Flores L. (2006). Risk for high-grade dysplasia or invasive carcinoma in colorectal flat adenomas in a Spanish population. *Gastroenterol. Hepatol.* 29 602–609. 10.1016/s0210-5705(06)71700-9 17198636

[B41] Pieri L. Vannucchi M. G. Faussone-Pellegrini M. S. (2008). Histochemical and ultrastructural characteristics of an interstitial cell type different from ICC and resident in the muscle coat of human gut. *J. Cell. Mol. Med.* 12 1944–1955. 10.1111/j.1582-4934.2008.00461.x 19145703 PMC4506162

[B42] Popescu L. M. (2011). The tandem: telocytes-stem cells. *Int. J. Biol. Biomed. Eng.* 5 83–92.

[B43] Popescu L. M. Faussone-Pellegrini M. S. (2010). Telocytes - a case of serendipity: the winding way from Interstitial Cells of Cajal (ICC), via Interstitial Cajal-Like Cells (ICLC) to telocytes. *J. Cell. Mol. Med.* 14 729–740. 10.1111/j.1582-4934.2010.01059.x 20367664 PMC3823108

[B44] Popescu L. M. Manole E. Serboiu C. S. Manole C. G. Suciu L. C. Gherghiceanu M. (2011). Identification of telocytes in skeletal muscle interstitium: Implication for muscle regeneration. *J. Cell Mol. Med.* 15 1379–1392. 10.1111/j.1582-4934.2011.01330.x 21609392 PMC4373336

[B45] Popescu L. M. Nicolescu M. I. (2013). “Telocytes and stem cells,” in *Resident Stem Cells and Regenerative Therapy*, eds Coeli dos Santos Goldenberg R. Campos de Carvalho A. C. (Waltham, MA: Academic Press/Elsevier), 205–231. 10.1016/b978-0-12-416012-5.00011-6

[B46] Pusztaszeri M. P. Seelentag W. Bosman F. T. (2006). Immunohistochemical expression of endothelial markers CD31, CD34, von Willebrand factor, and Fli-1 in normal human tissues. *J. Histochem. Cytochem.* 54 385–395. 10.1369/jhc.4A6514.2005 16234507

[B47] Ramaswamy A. Moll R. Barth P. J. (2003). CD34+ fibrocytes in tubular carcinomas and radial scars of the breast. *Virch. Arch.* 443 536–540. 10.1007/s00428-003-0855-6 12898244

[B48] Rusu M. C. Cretoiu D. Vrapciu A. D. Hostiuc S. Dermengiu D. Manoiu V. S. (2016). Telocytes of the human adult trigeminal ganglion. *Cell. Biol. Toxicol.* 32 199–207. 10.1007/s10565-016-9328-y 27147447

[B49] Shen Y. Wu Y. Zheng Y. Ao F. Kang K. Wan Y. (2016). Responses of adventitial CD34(+) vascular wall-resident stem/progenitor cells and medial smooth muscle cells to carotid injury in rats. *Exp. Mol. Pathol.* 101 332–340. 10.1016/j.yexmp.2016.11.004 27856167

[B50] Vannucchi M. G. Bani D. Faussone-Pellegrini M. S. (2016). Telocytes contribute as cell progenitors and differentiation inductors in tissue regeneration. *Curr. Stem Cell Res. Ther.* 11 383–389. 10.2174/1574888x10666150528142741 26018235

[B51] Vannucchi M. G. Traini C. Guasti D. Del Popolo G. Faussone-Pellegrini M. S. (2014). Telocytes subtypes in human urinary bladder. *J. Cell Mol. Med.* 18 2000–2008. 10.1111/jcmm.12375 25139461 PMC4244015

[B52] Vannucchi M. G. Traini C. Manetti M. Ibba-Manneschi L. Faussone-Pellegrini M. S. (2013). Telocytes express PDGFRα in the human gastrointestinal tract. *J. Cell. Mol. Med.* 17 1099–1108. 10.1111/jcmm.12134 24151977 PMC4118169

[B53] Vannucchi M. G. Faussone-Pellegrini M. S. (2016). “The telocyte subtypes,” in *Telocytes. Advances in Experimental Medicine and Biology*, Vol. 913, eds Wang X. Cretoiu D.(Singapore: Springer). 10.1007/978-981-10-1061-3_7 27796883

[B54] Wessel C. Westhoff C. C. Nowak K. Moll I. Barth P. J. (2008). CD34(+) fibrocytes in melanocytic nevi and malignant melanomas of the skin. *Virch. Arch.* 453 485–489. 10.1007/s00428-008-0667-9 18813945

